# A New and Rapid LC-MS/MS Method for the Determination of Cysteamine Plasma Levels in Cystinosis Patients

**DOI:** 10.3390/ph17050649

**Published:** 2024-05-16

**Authors:** Raffaele Simeoli, Sara Cairoli, Marcella Greco, Francesco Bellomo, Alessandro Mancini, Chiara Rossi, Carlo Dionisi Vici, Francesco Emma, Bianca Maria Goffredo

**Affiliations:** 1 Division of Metabolic Diseases and Hepatology, Bambino Gesù Children’s Hospital, IRCCS, 00165 Rome, Italy; raffaele.simeoli@opbg.net (R.S.); sara.cairoli@opbg.net (S.C.); alessandro.mancini@opbg.net (A.M.); chiara.rossi@opbg.net (C.R.); carlo.dionisivici@opbg.net (C.D.V.); 2 Division of Nephrology, Bambino Gesù Children’s Hospital, IRCCS, 00165 Rome, Italy; marcella.greco@opbg.net (M.G.); francesco.emma@opbg.net (F.E.); 3 Laboratory of Nephrology, Bambino Gesù Children’s Hospital, IRCCS, 00165 Rome, Italy; francesco.bellomo@opbg.net

**Keywords:** cystinosis, cysteamine, rapid assay, LC-MS/MS, therapeutic drug monitoring, pharmacokinetic (PK)

## Abstract

Cystinosis is a rare lysosomal storage disorder caused by autosomal recessive mutations in the *CTNS* gene that encodes for the cystine transporter cystinosin, which is expressed on the lysosomal membrane mediating the efflux of cystine. Cysteamine bitartrate is a cystine-depleting aminothiol agent approved for the treatment of cystinosis in children and adults. In this study, we developed and validated a liquid chromatography–tandem mass spectrometry (LC-MS/MS) method for the determination of cysteamine levels in plasma samples. This LC-MS/MS method was validated according to the European Medicines Agency (EMA)’s guidelines for bioanalytical method validation. An ultra-performance liquid chromatograph (UPLC) coupled with a 6470 mass spectrometry system was used for cysteamine determination. Our validated method was applied to plasma samples from n = 8 cystinosis patients (median, interquartile range (IQR) = 20.5, 8.5–26.0 years). The samples were collected before cysteamine oral administration (pre-dose) and 1 h after (post-dose). Our bioanalytical method fulfilled the regulatory guidelines for method validation. The cysteamine plasma levels in pre-dose samples were 2.57 and 1.50–3.31 μM (median and IQR, respectively), whereas the post-dose samples reported a cysteamine median concentration of 28.00 μM (IQR: 17.60–36.61). Our method allows the rapid determination of cysteamine plasma levels. This method was successfully used in cystinosis patients and, therefore, could be a useful tool for the evaluation of therapy adherence and for future pharmacokinetic (PK) studies involving a higher number of subjects.

## 1. Introduction

Cystinosis is a rare autosomal recessive lysosomal storage disease with an estimated incidence of 1:100,000–200,000 live births [1]. In its more frequent and severe form, termed infantile nephropathic cystinosis (OMIM 219800), patients present early in life with renal Fanconi syndrome and progressively develop a multisystem disorder related to cystine accumulation in virtually all organs, including the eyes, endocrine and reproductive organs, muscles, bones, lungs, skin, and the central nervous system [2]. The disease is caused by mutations in the *CTNS* gene, which encodes for cystinosin, a 367-amino acid lysosomal cystine-H+ symporter that mediates cystine efflux into the cytosol and interacts with other proteins, including the mTORC1 complex and vacuolar ATPase [1,3,4]. Currently, more than 100 pathogenic variants have been described in patients with infantile nephropathic cystinosis, without significant genotype–phenotype correlations [5]. The most frequent pathogenic variant in Northern Europe is a 57 kB deletion involving two neighboring genes, namely, the *CARKL* and *TRPV1* genes [6].

In most cells, cystine levels parallel their lysosomal endowment. In the blood, cystine accumulates more granulocytes and monocytes that are rich in lysosomes compared with lymphocytes [6]. Currently, the only established method for monitoring therapy in cystinosis relies on measuring cystine levels in peripheral leukocytes, preferably in purified granulocyte fractions [3]. Pharmacological treatment is based on lifelong oral administration of cysteamine, which was discovered in 1976 as a therapeutic agent for cystinosis [7] and is approved by the EMA and the FDA for the treatment of cystinosis. Over the years, cysteamine has been prescribed in different formulations, including cysteamine hydrochloride, phosphocysteamine, and cysteamine bitartrate [8]. Nowadays, most patients in developed countries are treated lifelong with cysteamine bitartrate, which is commercially available as short-acting (Cystagon^®^) or long-acting (Procysbi^®^) preparations that patients take orally every 6 or 12 h, respectively [9].

Cysteamine easily penetrates into the cytosol of cells and enters lysosomes through an unknown transporter [10]. In lysosomes, it interacts with cystine in a 1:1 stoichiometric reaction that breaks the disulfide bond of cystine and produces one molecule of cysteine and one molecule of cysteine–cysteamine for every molecule of cystine. The cysteine exits the lysosome through a cysteine transporter, while the mixed disulfide exits through the PQLC2 lysine transporter [1].

Since it was introduced for the treatment of patients with cystinosis, cysteamine has dramatically improved the prognosis of the disease and the life expectancy of patients. However, it cannot completely prevent all complications of the disease, including progression to end-stage kidney disease, and compliance is severely hampered by side effects, in particular, frequent gastrointestinal upset, unpleasant smells, and halitosis [11]. The monitoring of therapy aims at achieving sufficient cystine depletion while avoiding excessive dosages to limit side effects. To date, this is achieved by measuring leukocyte cystine levels, which have been shown to correlate with prognosis [5]. However, the measurement of leukocyte cystine levels is expensive and cumbersome [3]. Several pre-analytic steps, including sample storage and shipment, cell separation, cell sonication, and protein determination, introduce biases that limit the accuracy of measurements. Historically, leukocyte cystine measurements were used for diagnosing cystinosis and, thereafter, for monitoring therapy based on the observation that heterozygous carriers are asymptomatic despite their levels being 3–4 times higher than those measured in control subjects. When cysteamine was introduced into clinical practice, the technology did not allow easy measurements of cysteamine plasma levels and, in the absence of long-term studies that defined the optimal therapeutic range, assessing leukocyte cystine levels was preferred because it provided a target for adjusting doses (i.e., the levels in heterozygous carriers), However, these measurements are cumbersome and lack precision. With the advent of new technologies, cysteamine plasma level determination may represent an alternative strategy that avoids many of the pre-analytic hurdles of leukocyte cystine measurements and the shipment of stable frozen samples.

Steady-state pharmacokinetic and pharmacodynamic (PK/PD) studies on short- and long-acting cysteamine bitartrate performed in patients with nephropathic cystinosis have shown that 6 h and 12 h dosing intervals can maintain white blood cell cystine contents within the reference levels derived from heterozygous carriers [9,12,13,14,15,16]. These same studies have shown an inverse correlation between leukocyte cystine levels and cysteamine plasma concentrations. However, additional studies are needed to better define the PK/PD of cysteamine following oral treatment. In this context, population PK modeling (popPK) approaches followed by Monte Carlo simulations could be adopted not only to characterize cysteamine’s PK properties in specific patient populations but also to suggest tailored dosing regimens that are able to facilitate the achievement of target intra-leukocyte cystine concentrations [15].

So far, cysteamine levels have been measured using ultra-high-performance liquid chromatography coupled with tandem mass spectrometry (UHPLC-MS/MS) in different matrices, including human plasma [14,15,17,18,19,20]. Alternatively, in previous years, different analytical techniques have been proposed to detect and quantify cysteamine levels in various biological specimens [12,21,22,23].

Herein, we present a new LC-MS/MS-based method that allows the fast and reliable quantification of cysteamine levels in plasma samples. We validated this method in accordance with the ICH M10 guideline for bioanalytical method validation and study sample analysis [24] and tested it in samples obtained from patients with nephropathic cystinosis treated with cysteamine. In the future, this method could be used for therapeutic drug monitoring (TDM) to adjust cysteamine doses and to monitor patient compliance once the correlation between plasma cysteamine and leukocyte cystine levels has been better defined.

## 2. Results

### 2.1. Linearity

**Figure 1:**
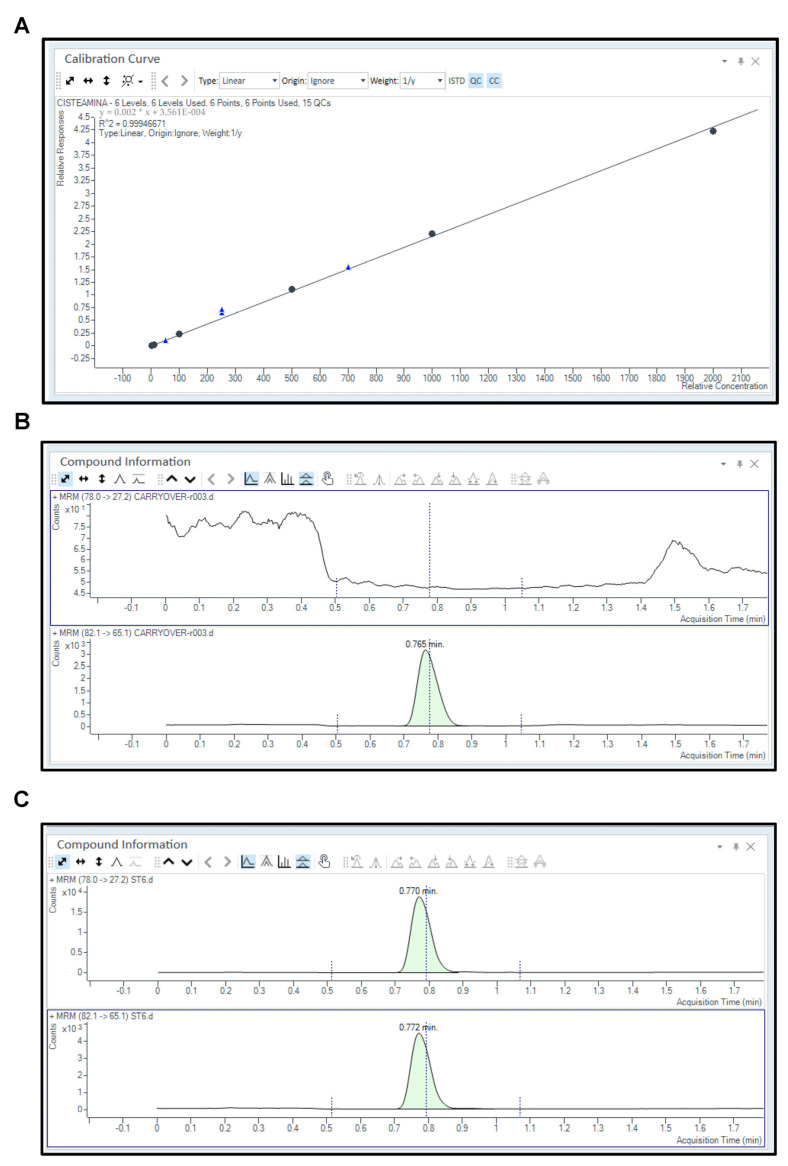
**Chromatograms obtained for cysteamine.** (**A**) Six-point calibration curve for cysteamine. The calibration curve equation and R2 value are displayed in the inset. Blue triangles indicate L-QC, M-QC, and H-QC samples. (**B**) Chromatograms of blank plasma sample spiked with deuterated IS. (**C**) Chromatograms of plasma calibration point 6 (ST6). For each chromatogram, the upper and lower layers indicate the fragments used as the quantifier and the internal standard compound, respectively. The relative response (counts) from the baseline and the acquisition time (min) are reported on the y- and x-axes, respectively. For each peak, the retention time is displayed.

A weighted 1/y calibration curve was used to cover the following concentrations: 1, 10, 100, 500, 1000, and 2000 µM (R^2^ = 0.9994; y = 0.00216 × x + 3.561 × 10^−4^) (Figure 1A). To further evaluate the linearity of the calibration curve, back-calculated concentrations for cysteamine calibration standards were assessed and the relative error (expressed as % bias) was also computed by comparing calculated with nominal concentrations. For each calibration standard, the % bias was within the acceptable value of ≤15%.

### 2.2. Accuracy and Precision

The intra- and inter-assays’ accuracy and precision were evaluated for L-QC, M-QC, and H-QC (Table 1 and Table 2). Both parameters agreed with the EMA guidelines for the validation of bioanalytical methods. Specifically, the intra- and inter-assays’ accuracy (reported as the mean %bias) was ≤15% at each QC level. Similarly, the precision (expressed as the %CV) was ≤15% for both the intra- and inter-assays at the low, medium, and high-QC levels.

### 2.3. Selectivity and Specificity

Drug-free plasma samples were spiked with or without cysteamine-D4 used as an internal standard (IS) and were analyzed to evaluate the possible endogenous interferences with cysteamine detection. As shown in Figure 1B, the blank samples spiked with the IS did not show interference peaks within the cysteamine chromatogram. Conversely, in Figure 1C, the chromatogram relative to calibrator 6 (ST6; 2000 µM) shows the peaks for cysteamine (upper layer) and cysteamine-D4 (IS) (lower layer) at a retention time (rt) of 0.77 min (mins).

However, the median signal of these blank samples was below 20% of the LLOQ, thereby ensuring the selectivity of the method.

In terms of chromatographic separation, this was realized with an InfinityLab Poroshell 120 HILIC 1.90 μm (100 × 2.1 mm) column. Based on a dead volume of around 150 μL and a flow rate of 0.5 mL/min, our calculated dead time (t_0_) was around 0.25–0.30 min. Therefore, also considering that cysteamine is a highly polar molecule, in our study, the retention (or capacity) factor (k), calculated as the ratio of the rt of the analyte in the column (0.77 min) to the rt of a non-retained compound (0.30 min), was between 1.5 and 2.0, highlighting a good separation. In fact, k-values > 1 indicate that the analyte has been retained and has spent a significant amount of time interacting with the stationary phase. Indeed, the value recorded was sufficient to guarantee the detection of both cysteamine and cysteamine-D4 in the defined rt window.

The LLOQ concentration was 0.50 µM and was determined by dissolving decreasing concentrations of cysteamine powder in pooled drug-free plasma. The LLOQ was identified and confirmed with an accuracy and precision within 20% (Supplementary Figure S1). Moreover, the intra- and inter-assays’ accuracy and precision at the LLOQ level were measured from the six-point calibration curve. The results are reported in Table 1 and Table 2.

In order to assess the presence of carry-over, IS-spiked blank samples were run in triplicate, following the highest calibration point. In agreement with the EMA guidelines, the median signal of these blank samples was less than 20% of the LLOQ and 5% of the IS, confirming the absence of carry-over (Figure 1B).

Analyses performed to evaluate the matrix effect and IS-normalized matrix effect showed values within the acceptable range (80–90%) for cysteamine measurements. Similarly, the extraction recovery (%ER) was between 82 and 90%. The results for both the ME% and RE% are displayed in Table 3.

Moreover, to further assess the matrix effect, we also used pooled plasma from n = 6 different cysteamine-treated patients. Thereafter, the post-extraction matrix was spiked with cysteamine-D4 in order to discriminate the spiked cysteamine from that already present in the matrix (B). The same amount of cysteamine-D4 was spiked in a pure solution (A). The ME% was calculated as B/A × 100% (as already described in the Section 4) and resulted in a percentage slightly higher than 100% (i.e., 108%). Therefore, we can conclude that the presence of cysteamine in patients’ samples does not affect ME evaluation.

### 2.4. Measurement of Cysteamine Plasma Levels in Patients with Infantile Nephropathic Cystinosis

**Figure 2:**
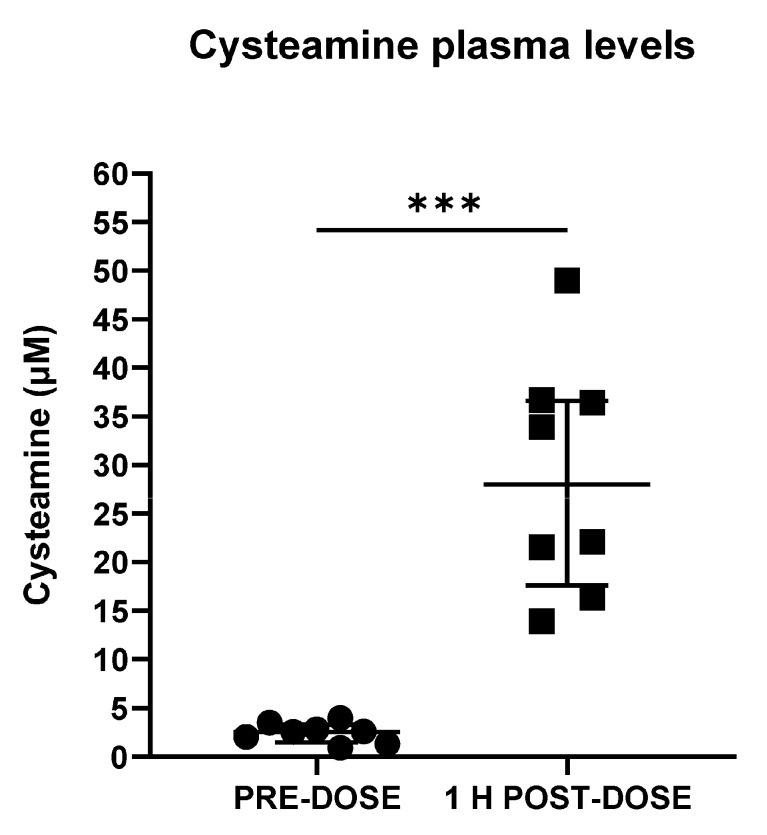
**Cysteamine plasma levels.** Box plots of cysteamine plasma levels measured in n = 8 cystinosis patients before (pre-dose) and 1 h after (post-dose) single-dose oral administration. Medians and interquartile range (IQRs) are displayed. *** *p* < 0.001 vs. pre-dose.

To test the clinical applicability of the method, cysteamine levels were measured in the plasma samples from eight patients (median, IQR = 20.5, 8.5–26.0 years) treated with cysteamine obtained before (pre-dose) and 1 h after short-acting cysteamine bitartrate oral administration (post-dose). The patient characteristics are reported in Table 4. After treatment, the cysteamine plasma levels increased from a median value of 2.57 (IQR: 1.50–3.31 μM) to 28.00 μM (IQR: 17.60–36.61) (Figure 2).

### 2.5. Evaluation of Short- and Long-Term Autosampler Stability

Both short- and long-term stability were evaluated in prepared QC samples kept at room temperature for a maximum of 19 days. After 24 h from day 0 (the QC sample preparation and first assessment), the stability was around 90% for the medium- and high-QC samples (>50 μM). The cysteamine concentrations were not quantifiable for the low-QC samples. After 48 h, the stability dropped dramatically and was no longer detectable (nd) in the low- and medium-QC samples (Table 5). Similar results were observed after 7 and 19 days.

## 3. Discussion

Since its introduction into clinical practice, cysteamine has dramatically improved the prognosis of cystinosis. However, treatment monitoring has remained cumbersome and is currently based on the clinical evaluation of side effects and measuring intra-leukocyte cystine levels [25]. If possible, measurements should be performed on purified granulocyte fractions since these cells are more rich in lysosomes [6]. Currently, leukocyte cystine levels are the gold standard for monitoring therapy and have been shown in a large retrospective cohort to correlate with kidney function outcomes [3,5]. In most laboratories, the target levels are <1 nmol cystine/mg protein; however, these can vary depending on the methodology that is used in individual laboratories, on the type of anticoagulation adopted for blood drawing, on the leukocyte fraction that is analyzed (the whole leukocyte pool or purified granulocytes), and on the method that is used for measuring proteins [3]. Currently, only a handful of laboratories perform this test worldwide, and, therefore, samples frequently need to be shipped, which represents an additional disadvantage, since they cannot be frozen before the pre-analytical steps are carried out on living cells. Hence, shipments must guarantee the delivery of samples usually within 24 h, and samples need to be processed immediately upon arrival in the laboratory bench. In addition, the shipment temperature may be a critical aspect during hot seasons. The combined result of all these limitations is that measurements of leukocyte cystine levels are notoriously variable and physicians often wait two or three consecutive measurements before implementing therapy. 

Theoretically, it could be advantageous to directly measure cysteamine concentrations in plasma [14]. In fact, this does not involve the same preanalytical steps and can be performed on frozen plasma samples that can be more easily shipped. Moreover, previous studies have already shown an inverse correlation between cysteamine plasma levels and intra-leukocyte cystine contents [9,12,13,14,15,16]. 

Herein, we established a new method that allows measuring cysteamine plasma concentrations rapidly and accurately. When samples were analyzed within 24 h of storage at room temperature, this method fulfilled all the quality criteria for accuracy, precision, selectivity, specificity, the carry-over effect, the matrix effect, and the appropriateness of sample recovery. 

In the era of personalized medicine, the role of TDM extends beyond the simple measurement of drug levels and has become a comprehensive approach that allows reaching therapeutic efficacy without unnecessary toxicity [26]. This includes testing for therapy adherence, which is particularly relevant in lifelong treatments that have significant side effects, such as cysteamine therapy. In fact, the strict dosing regimen based on immediate-release (IR) cysteamine bitartrate (Cystagon^®^) requires intake every 6 h, even during the night [9]. This aspect, together with various adverse side effects, like body odor and halitosis, nausea, vomiting, and diarrhea, leads to poor therapy adherence and compliance problems [9]. Moreover, undesired effects such as Ehlers−Danlos syndrome, elevated alkaline phosphatase, ulcers/bleeding in the gastrointestinal tract, and idiopathic intracranial hypertension (IIH), which can cause ringing in the ears, loss of vision, or pain due to eye movement along with dizziness, have been also described [21]. Therefore, the TDM of cysteamine could be a viable tool for monitoring plasma concentrations in order to guarantee therapeutic levels and avoid toxic effects. 

In future studies, monitoring cysteamine plasma levels in combination with leukocyte cystine levels will allow for defining the best time points for extrapolating the parameters of cysteamine PK exposure that correlate best with cystine depletion. Perhaps, the use of nonlinear mixed-effects approaches for population PK modeling (popPK) followed by Monte Carlo simulations could be adopted not only to characterize cysteamine’s PK properties in specific patient populations but also to suggest tailored dosing regimens that are able to facilitate the achievement of target intra-leukocyte cystine concentrations [15].

As a proof of concept, in the present study, we applied an LC-MS/MS-based methodology to samples obtained from patients treated with cysteamine. Our results show that this method can clearly detect the expected rise in cysteamine concentrations after oral intake and that measured levels are in agreement with previous data obtained in pediatric patients [12]. So far, cysteamine has been measured by using UHPLC-MS/MS technology in different matrices including human plasma [14,15,17,18,19,20]. However, in the last decades, alternative bioanalytical methods have been proposed to measure cysteamine levels in various biological specimens. These include high-performance liquid chromatography (HPLC) with fluorescence and ultraviolet (UV) detection [12,22], a chromogenic sensor for cysteamine based on the azophenol−Cu^2+^ complex system [21], and colorimetric assays using peroxidase tablets that are able to react with cysteamine in artificial and real human serum samples [23]. Additionally, methods based on high-voltage electrophoresis, ion-exchange column chromatography, and gas chromatography with flame ionization and photometric detection have also been reviewed by Atallah C. and colleagues (2020) [27].

Although these methods often rely on more accessible technologies and are characterized by the advantages of simplicity, versatility, and cost-effectiveness, they show less sensitivity and selectivity compared with LC-MS/MS. In fact, absorbance-based quantitative analyses present limitations in colorimetric assays, including the presence of interfering substances in samples, limited dynamic ranges, and a lack of specificity that leads to cross-reactivity with undesired compounds. Additionally, these methods are often characterized by prolonged incubation times that, alongside the sample preparation and analytical run duration, make the turnaround time (TAT) undesirable for a biochemistry laboratory working within routine clinical practice. 

It is also worth saying that due to the lack of a chromophore in the cysteamine structure, the quantification of this compound using conventional analytical methods with UV absorbance or fluorescence detection presents some challenges. Therefore, the derivatization of cysteamine is used for its separation or quantification. For this purpose, several derivatization agents have been proposed and optimized according to the analytical method applied [27]. 

Moreover, the detection of cysteamine in plasma presents additional challenging aspects. Apart from cysteamine’s PK properties (low absorptivity), its susceptibility to oxidation (before or during analysis) and reactivity with other endogenous thiol moieties represent additional hurdles [27]. Specifically, cysteamine can bind to cysteine residues of plasma proteins or other endogenous aminothiols such as cysteinylglycine, homocysteine, glutathione, and γ-glutamylcysteine [28], leading to the formation of disulfide crosslinks [29]. The determination of free cysteamine in plasma requires reducing disulfides prior to the analysis. Several reducing agents have been proposed, including dithiothreitol (DTT) and Tris(2-carboxyethyl) phosphine (TCEP) [14,30,31]. In the present study, in order to selectively and quantitatively reduce disulfide bonds, we used TCEP as previously described in [31]. Optimal TCEP-reducing conditions for measuring total biothiols in mouse serum samples have been previously described in [30,32,33]. These studies showed that TCEP reduces disulfide bonds as effectively as dithiothreitol (DTT) but offers additional advantages because it is non-volatile, odorless, and, unlike most other reducing agents, is resistant to air oxidation. It selectively and completely reduces even stable water-soluble alkyl disulfides over a wide pH range. Moreover, unlike DTT and other thiol-containing reducing agents, TCEP does not have to be removed during sample preparation (https://www.biosyn.com/, accessed on 4 September 2023).

Differently from previously published protocols, our method uses deuterated cysteamine as an internal standard (cysteamine-D4) and can be performed in very low sample volumes (50 µL). This latest aspect is particularly valuable and “makes our sample preparation applicable to both adults and pediatric patients with cystinosis, including infants and small children for whom microsampling devices and capillary blood sampling can be also used to monitor their condition (in particular their renal Fanconi syndrome) through multiple blood tests”.

Moreover, in order to prevent the risk of oxidation, blood samples were collected in EDTA-containing tubes, as EDTA seems to play a role in preventing cysteamine oxidation during the pre-analytical phase [34]. Additionally, light-dependent oxidation during sample preparation was prevented by using amber tubes for both stock and working solutions.

Concerning deuterated internal standards in LC-MS/MS, it is well recognized that using an isotopically labeled version of the analyzed molecule represents an advantageous condition and provides significant robustness to the bioanalytical method. 

Additionally, our chromatographic run time is short (7.5 min). This aspect compensates for the TCEP incubation period and allows us to establish an acceptable turnaround time, which is an important prerequisite for the usefulness of TDM. 

Although the cysteamine rt is 0.77 min, the choice of using gradient elution and prolonging the run time up to 7.5 min allows better separation between different analytes, especially in the presence of potential interference peaks within the target rt window. In our chromatographic separation procedure, the gradient conditions were chosen to facilitate an adequate column re-equilibration at each injection. As a consequence, the same initial conditions in terms of column pressure were newly guaranteed for every sample’s injection.

Furthermore, our method is characterized by an easy sample preparation, as previously described in [31]. In fact, apart from deuterated IS and TCEP, the plasma samples are not enriched with derivatizing chemical agents [14,19] and/or cysteine protease inhibitors (e.g., N-ethylmaleimide or NEM) [14,15]. Finally, in our method, a time-consuming supernatant evaporation step is not required [14].

However, Supplementary Table S1 shows the main differences in the LC-MS/MS parameters between our method and previously published reports on cysteamine quantification in plasma samples [14,15,19].

Our method was fully validated in accordance with the most recent EMA guidelines for bioanalytical methods in terms of accuracy, precision, selectivity, specificity, the absence of carry-over, the matrix effect, and recovery [24]. The autosampler stability was evaluated in low-, medium-, and high-QC samples kept at room temperature for up to 19 days. The stability results indicate that once prepared, samples must be analyzed within 24 h. This aspect also represents an improvement compared with previous studies that have evaluated the stability of cysteamine in plasma by analyzing working solutions (100 µM) used for the preparation of calibrators/QC and stored for up to 3 months at −20 °C [14]. Following bioanalytical method validation, in order to test the applicability of our method to clinical samples, cysteamine levels were measured in plasma specimens collected from cystinosis patients before (C*trough*) and 1 h after (C*max*) the administration of one daily dose of Cystagon^®^. The time to reach the maximum plasma concentration (T*max*) was chosen according to previously described evidence (range: 1.0–2.0 h) [12]. Both exposure PK parameters agreed with those reported in a PK/PD study conducted by Belldina E. B. and colleagues on cysteamine bitartrate use in pediatric cystinosis patients [12]. Similarly, in our study, the median cysteamine concentration for C*max* (post-dose) was 28.00 µΜ (IQR: 17.62–36.61), corresponding to 2160.20 ng/mL. This value was slightly higher than the peak concentration reported in a phase I pharmacokinetic study on cysteamine bitartrate conducted in adult patients (C*max* range: 1700.0–2000.0 ng/mL) [35]. However, in the study by Armas D. and colleagues, the use of delayed-release capsules of cysteamine bitartrate produced a mean T*max* between 2.50 and 3.50 h [35]. Therefore, the use of a different oral formulation could partially explain the difference observed in our C*max* values. Conversely, the median C*max* measured in our study was comparable to those reported in previously published PK studies performed on adult subjects being administered different cysteamine bitartrate formulations [36,37].

It is also worth saying that the potential limitations of this study include the small number of clinical samples used to test the method’s applicability (partially due to the rare incidence of cystinosis) and the absence of analyses of the correlation between cysteamine plasma levels and intra-cellular cystine contents performed for each patient at both sampling time points. 

However, our aim herein was not to realize a PK study but to develop and validate a rapid and sensitive LC-MS/MS-based bioanalytical method that could be used to monitor cysteamine therapy in nephropathic patients. Future studies involving a larger number of patients will be needed to define not only a therapeutic range for cysteamine in both adult and pediatric patients but also to assess the relationship between intra-cellular cystine contents and cysteamine plasma concentrations. 

In conclusion, we developed a new method for fast and simple measurements of cysteamine in low plasma volumes that can be easily adopted to conduct PK/PD studies. This will allow accurate assessments of drug exposure in children treated with short- and long-acting cysteamine bitartrate formulations, aiming to guarantee therapeutic plasma concentrations and avoid the occurrence of undesired adverse events. Finally, by establishing a simpler analytical methodology for the therapeutic drug monitoring of cysteamine, future research will aim to replace less convenient methods for measuring intra-leukocyte cystine levels in diagnostic laboratories in routine clinical practice.

## 4. Materials and Methods

### 4.1. Chemicals and Reagents

Acetonitrile and formic acid were purchased from Biosolve Chemicals (Dieuze, France). Tris(2-carboxyethyl) phosphine hydrochloride (TCEP) and cysteamine were provided by Sigma-Aldrich (St. Louis, MO, USA). LC-MS-grade water was purchased from VWR International (Radnor, PA, USA). Cysteamine-D4 hydrochloride was purchased from LGC (Middlesex, UK).

### 4.2. Stock and Working Solutions

A 100 µM TCEP solution was prepared by dissolving 2.8 mg of TCEP powder in 100 mL of LC-MS-grade water. The TCEP stock solution was stored at +4 °C until use. A cysteamine stock solution was prepared at a concentration of 10 mM by dissolving 7.50 mg of analyte in 10 mL of LC-MS-grade water. Similarly, a stock solution of cysteamine-D4 hydrochloride (used as an internal standard) was prepared at 1 mg/mL in LC-MS-grade water. Both the cysteamine and cysteamine-D4 stock solutions were stored at −80 °C until use. A working solution of cysteamine-D4 was prepared alongside the sample preparation by diluting the stock solution to 1:100 in LC-MS-grade water. Amber tubes were used for both the stock and working solutions in order to avoid light-dependent oxidation.

### 4.3. Calibration Standards and Quality Control Samples

A six-point calibration curve (excluding blank samples) was obtained by performing serial dilutions from the cysteamine stock solution (10 mM) in drug-free plasma pooled from different healthy donors. The calibrator (CAL) concentrations were as follows: 1, 10, 100, 500, 1000, and 2000 µM. Samples above the highest calibration point were further diluted using pooled blank plasma. Similarly, n = 3 quality controls (QC) were prepared from the cysteamine stock solution at 50, 333, and 714 µM for low-, medium-, and high-QC levels (L-QC, M-QC, and H-QC), respectively. The lower limit of quantification (LLOQ) was 0.5 µM and was determined by dissolving decreasing concentrations of cysteamine powder in pooled drug-free plasma. Thereafter, n = 40 replicates in five different analytical sessions were injected and analyzed. The LLOQ was identified and confirmed with an accuracy and precision of up to 20%. 

### 4.4. Human Samples

Drug-free plasma samples were collected from healthy donors recruited at the Blood Transfusion Center of the Bambino Gesù Children’s Hospital (Rome, Italy) after obtaining informed consent and were used as biological matrices for preparing the LLOQ, calibration standards (CALs), low-, medium-, and high-quality controls (QCs), and blank samples to assess selectivity and specificity. The drug-free plasma samples were pooled and stored at −20 °C until use.

The cysteamine plasma levels were measured in 8 samples obtained from patients suffering from nephropathic cystinosis who were treated at the Bambino Gesù Children’s Hospital in Rome. In order to evaluate the pharmacokinetic (PK) exposure parameters in a steady state, the trough (C*trough*) and maximal (C*max*) concentrations were measured by collecting EDTA–whole blood samples before the next dose (C*trough*) and 1 h after administration (C*max*). Thereafter, the plasma was recovered from the EDTA–whole blood samples by centrifugation at 3500 rcf for 5 min. The patients’ plasma samples were stored at −80 °C until processing.

All cystinosis patients enrolled in this study were treated with short-acting cysteamine (Cystagon^®^ capsules). Informed consent was obtained from adult patients or the parents of patients aged less than 18 years. Since this study was non-interventional, the Bambino Gesù Children’s Hospital Ethics Committee was only informed in writing. All analyses were performed after sample anonymization. 

### 4.5. Determination of Cysteamine Plasma Levels by LC-MS/MS

This study was conducted in the Laboratory of Metabolic Diseases and Drug Biology of the Bambino Gesù Children’s Hospital in Rome (Italy). Cysteamine plasma levels were determined by high-performance liquid chromatography (HPLC) coupled with mass spectrometry (MS/MS). EDTA-containing whole blood samples were collected before oral cysteamine administration (C*trough*) and after 1 h (C*max*). Plasma was recovered by centrifugation at 3500× *g* for 5 min and stored at −80 °C until processing. Liquid chromatography (LC) was performed with a UHPLC Agilent 1290 Infinity II apparatus (Agilent Technologies, Deutschland GmbH, Waldbronn, Germany). Chromatographic separation was performed with an InfinityLab Poroshell 120 HILIC 1.90 µm (100 × 2.1 mm) column (Agilent Technologies) maintained at 30 °C. The mobile phase was delivered at a flow rate of 0.5 mL/min through gradient elution and consisted of 0.1% formic acid in milli-q pure water (aqueous mobile phase A) and 0.1% formic acid in acetonitrile (ACN; organic mobile phase B). The analytical run time for each injection was 7.50 min. The gradient conditions are reported in Supplementary Table S2. 

The injection volume was 10.0 μL. The detection of cysteamine and IS (cysteamine-D4) was based on the peak mass-to-charge (*m*/*z*) ratio and was performed with a 6470 mass spectrometry system (Agilent Technologies) equipped with an ESI-JET-STREAM source operating in positive ion (ESI+) mode. The mass spectrometric conditions were as follows: a gas temperature of 150 °C, a gas flow of 10 l/min, a sheath gas temperature of 400 °C, a sheath gas flow of 10 l/min, a 2000 V capillary, and a 40 psi nebulizer. The samples were detected in multiple-reaction monitoring (MRM) mode. The mass transitions for cysteamine were as follows: m/z 78.04 → 35.1 for the quantifier and 78.04 → 27.2 for the qualifier (Supplementary Figure S2). The mass transitions for cysteamine-D4 were m/z 82.07 → 30.2 for the quantifier and 82.07 → 65.1 for the qualifier. The MassHunter software v.10.1 (Agilent Technologies) was used for operating the system and analyzing the results. 

### 4.6. Sample Preparation

Briefly, 50 µL of the cysteamine-D4 working solution (used as an internal standard (IS)) was added to 50 µL of the CAL, QC, or plasma sample and 100 µL of the TCEP solution (100 µM) in amber tubes. The samples were mixed by vortexing for 30 s and were incubated at 38 °C for 60 min. Next, protein precipitation was carried out by adding 200 µL of acetonitrile (ACN). After mixing for 30 s and centrifugation at 13,000 rpm for 9 min at room temperature, 200 µL of the supernatant from each tube was transferred to a vial and injected into the UHPLC-MS/MS system for analysis.

### 4.7. Bioanalytical Method Validation

The method was validated according to the ICH M10 guideline on bioanalytical method validation and study sample analysis (25 July 2022 EMA/CHMP/ICH/172948/2019, the Committee for Medicinal Products for Human Use) (available at https://www.ema.europa.eu/en/ich-m10-bioanalytical-method-validation-scientific-guideline, accessed on 28 August 2023). Specifically, the accuracy, precision, selectivity, specificity, and presence of carry-over were evaluated. In addition, we also assessed the matrix effect and the recovery and stability of the samples. For all experiments, the acceptance criteria were set at ≤15% (≤20% at the LLOQ) for precision (expressed as the %coefficient of variation (CV)) and at ≤15% for accuracy (expressed as the mean %bias) (≤20% at the LLOQ).

#### 4.7.1. Accuracy and Precision

The intra- and inter-assays’ accuracy and precision were determined from 10 independent runs for each QC level over a period of four months. The accuracy was reported as the mean % bias; the precision was defined as the %coefficient of variation (CV) for the low-, medium-, and high-QC levels and the LLOQ.

#### 4.7.2. Selectivity and Specificity

The selectivity and specificity were assessed by confirming the absence of interference in drug-free plasma samples spiked with or without an internal standard. The median signal of blank samples should be <20% of the LLOQ to ensure the selectivity of the method. 

#### 4.7.3. Carry-Over

The presence of carry-over was evaluated by injecting blank plasma samples in triplicate after the highest calibration standard. According to EMA guidelines, carry-over is considered negligible if the signal in blank samples is less than 20% of the LLOQ and 5% for the IS.

#### 4.7.4. Matrix Effect and Extraction Recovery

The matrix effect and extraction recovery were evaluated in low-, medium-, and high-QC samples analyzed in triplicate. The matrix effect (ME) and extraction recovery (ER) were assessed by analyzing n = 6 different pools of blank matrix samples obtained from healthy donors. The matrix effect was calculated as B/A × 100%, where B represents the peak area of each analyte spiked into a blank matrix extract (spiked after extraction), and A is the peak area of each analyte in a pure solution at the same concentration [38]. The extraction recovery was calculated as C/B × 100%, where C is the peak area of each analyte spiked into a blank matrix before extraction. An ER% between 90 and 110% was considered acceptable. The ME% and RE% were also normalized for a deuterated internal standard (IS-normalized). 

#### 4.7.5. Stability

The stability was assessed by analyzing the cysteamine concentrations in QCs stored in an autosampler kept at room temperature on day 0 (the QC sample preparation and first assessment) and after 24, 48 h (short-term stability), or 19 days (long-term stability). The percentage difference was calculated as the ratio of the concentration measured at each sampling point to the initial concentration. According to EMA guidelines, the stability is considered acceptable if the percentage difference is lower than 15%.

### 4.8. Statistical Analysis

All statistical analyses were performed using GraphPad Prism v.9 (GraphPad Software, San Diego, CA 92108, USA). The demographic data and PK parameters were analyzed using descriptive statistics. Medians with interquartile ranges (IQRs) were used to describe C*trough* and C*max* values. The Mann–Whitney test was used as a nonparametric test to compare two groups of data. Statistical significance was set at *p* < 0.05.

## Figures and Tables

**Table 1:** Intra-assay’s accuracy and precision.

Parameter	Cysteamine			
Quality control sample (target concentration)	LLOQ (0.50 µM)	L-QC (50 µM)	M-QC (333 µM)	H-QC (714 µM)
**Number of analyzed samples**	10	10	10	10
**Cysteamine concentration found µg/mL (median, range)**	0.40 (0.43–0.55)	51.14 (50.39–51.74)	334.89 (302.03–336.44)	722.10 (720.65–726.24)
**Intra-assay %bias**	−4.8	2.0	−1.3	1.2
**Intra-assay %CV**	10.2	1.1	4.5	0.3

**Table 2:** Inter-assay’s accuracy and precision.

Parameter	Cysteamine			
Quality control sample (target concentration)	LLOQ (0.50 µM)	L-QC (50 µM)	M-QC (333 µM)	H-QC (714 µM)
**Number of analyzed samples**	10	10	10	10
**Cysteamine concentration found µg/mL (median, range)**	0.53 (0.43–0.60)	54.67 (51.02–55.95)	344.48 (328.75–358.36)	728.38 (665.24–745.02)
**Inter-assay %bias**	4.0	8.1	3.3	0.4
**Inter-assay %CV**	14.0	4.2	4.4	5.0

**Table 3:** Results of matrix effect (ME) and extraction recovery (ER) experiments.

	L-QC (50 µM)		M-QC (333 µM)		H-QC (714 µM)	
	ER%	ME%	ER%	ME%	ER%	ME%
**Analyte: cysteamine**	82	86	83	88	90	83
**Number of analyzed samples**	3	3	3	3	3	3

**Table 4:** Demographic characteristics of study patients and doses administered.

Patient ID	Age (Years)	Gender	Race	Weight (kg)	Height (cm)	Cysteamine (mg) *
1	8	M	Caucasian	16.7	110.6	700
2	18	F	Caucasian	47	146	1900
3	5	F	Caucasian	16.5	103.5	300
4	10	M	Caucasian	25.7	120	1100
5	26	F	Caucasian	39.5	152	2000
6	23	F	Caucasian	71	165.5	2000
7	38	M	Caucasian	63.5	160	2000
8	26	M	Caucasian	75.50	167.20	2400

* Daily dosing of cysteamine was four times a day.

**Table 5:** Short-term autosampler stability.

	Short-Term Stability		
Time Point	Day 0	Day 1	Day 2
**Measured concentration for** **L-QC (50 µM)**	56.62	nd	nd
**Stability (%)**	**100**	**nd**	**nd**
**Measured concentration for** **M-QC (333 µM)**	361.52	327.56	nd
**Stability (%)**	**100**	**94**	**nd**
**Measured concentration for** **H-QC (714 µM)**	673.13	666.10	396.80
**Stability (%)**	**100**	**90**	**54**

## Data Availability

The data will be made available on request.

## Supplementary Materials

The following supporting information can be downloaded at https://www.mdpi.com/article/10.3390/ph17050649/s1, Supplementary Figure S1. Overlaid chromatograms of blank plasma alone and spiked at LLOQ level; Supplementary Figure S2. Mass spectrum results; Supplementary Table S1. Comparison between bioanalytical method described here and previous published LC-MS/MS methods; Sup-plementary Table S2. Gradient conditions reached during the chromatographic separation.
