# Supplementary material for: A New and Rapid LC-MS/MS Method for the Determination of Cysteamine Plasma Levels in Cystinosis Patients
Source: Pharmaceuticals (Basel). 2024 May 16;17(5):649. doi: 10.3390/ph17050649 (PMC11124818; doi:10.3390/ph17050649)
Supplement: Supplementary file 1 [file pharmaceuticals-17-00649-s001.zip › pharmaceuticals-2892830-supplementary.pdf]

Supplementary Figure S1 Overlaid chromatograms of blank plasma alone and spiked at LLOQ level

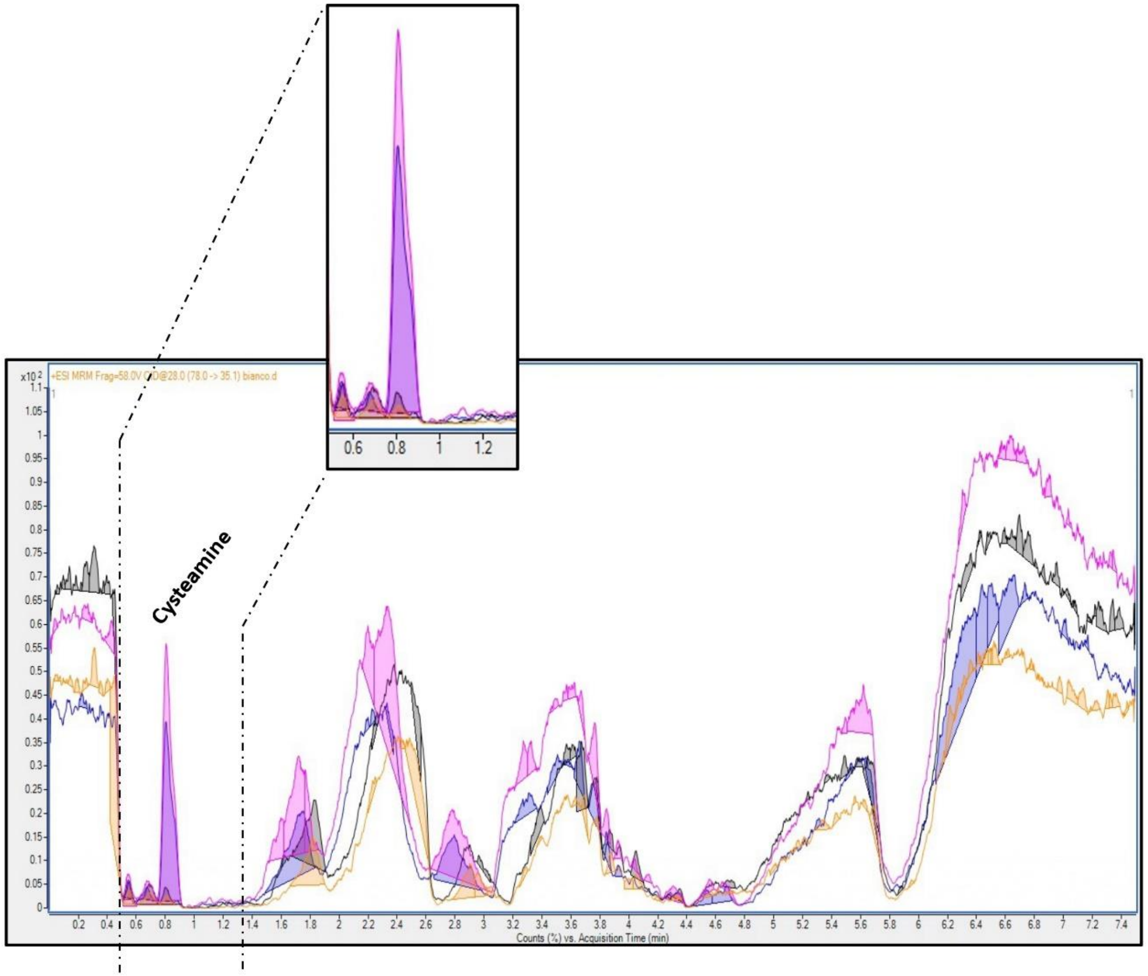

Supplementary Figure S2 Mass spectrum results

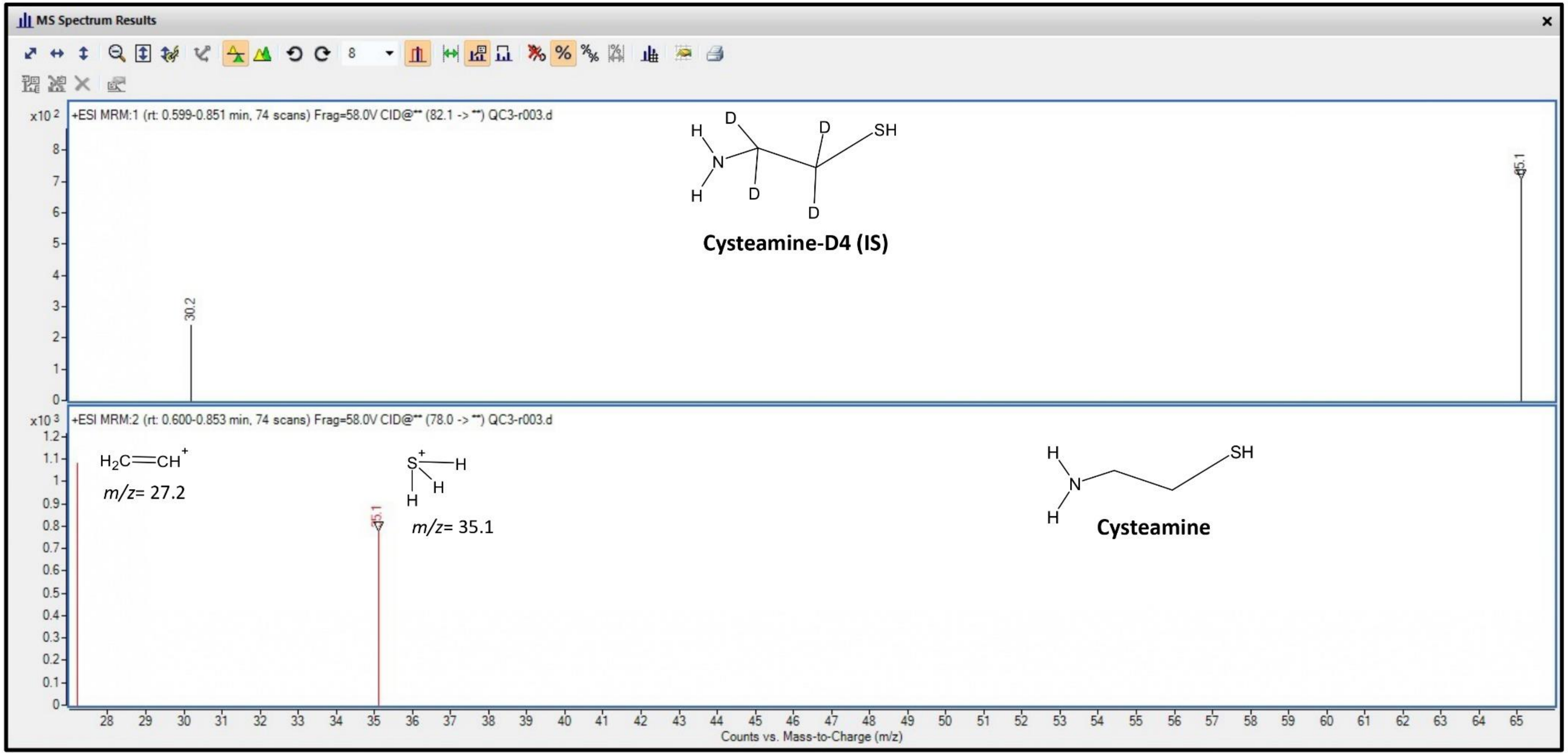

Supplementary Table 1. Comparison between bioanalytical method described here and previous published LC-MS/MS methods.

Bioanalytical method	Instrumentation	Chromatographic column	Elution mode and Mobile phases used	Injection volume	Flow- rate (mL/min)	Run-time	Analysed matrix (required volume)	Disulfide-Reducing Agent	Calibration range; LLOQ	Internal standard (IS) used
Method described here	UHPLC Agilent 1290 Infinity II apparatus (Agilent Technologies) coupled with 6470 Mass Spectrometry system (Agilent Technologies)	InfinityLab Poroshell 120 HILIC 1.90 µm (100 x 2.1 mm)	Gradient elution.<br><br>Mobile phase A (0.1 % formic acid in milli-q pure water);<br><br>Mobile phase B (0.1% formic acid in acetonitrile ACN);	10.0 µL	0.5	7.50 minutes	Plasma (50 µL)	Tris (2-carboxyethy) phosphine (TCEP)	1-2000 µM; 0.50 µM	D4-(deutered) cysteamine
Dohil R. et al., 2005 [19]	API 2000 LC-MS/MS, (Applied Biosystems)	LC-CN 2 cm x 4.0 mm I.D. 3 µm particle size (Supelco)	Isocratic elution.<br><br>Mobile phase (0.1% formic acid)	20.0 µL	0.4	Not reported	Plasma (100 µL)	Tris (2-carboxyethy) phosphine (TCEP)	1-100 µM	2-dimethyl-ethane-aminothiol

Bouazza N. et al., 2011 [15]	Agilent 1100 system (Agilent Technologies) coupled to Applied Biosystems/MDS Sciex API3000 MSMS	XD8-C8 column 150 × 4.6 mm (Interchim)	Isocratic elution. Mobile phase [water/acetonitrile (410/90) with 0.1% formic acid].	5.0 µL	0.6	Not reported	Plasma (200 µl)	Dithiothreitol (DTT)	Not reported	D4-(deutered) cysteamine
Franzin M et al., 2022 [14]	SCIEX ExionLCTM AD (AB Sciex) coupled with the mass spectrometer SCIEX Triple QuadTM 6500 + LC-MS/MS System	Poroshell Agilent 120 EC-C8 150 mm × 2.1 mm, 4 µm (Agilent Technologies)	Gradient elution. Mobile phase A (water with 0.15% formic acid and 5 mM ammonium formate). Mobile phase B (acetonitrile: water 95:5 with 0.15% formic acid and 5 mM ammonium formate).	3.0 µL	0.4	14 minutes	Plasma (100 µL)	Dithiothreitol (DTT)	2.5-50 µM; 1.25 µM	D6-(deutered) cystine

For reference numbers see the main manuscript.

Supplementary Table 2. Gradient conditions reached during the chromatographic separation.

Time (min)	Mobile Phase B (%)
0	78
1	78
4	20
4.5	20
4.6	78
7.5	78
